# Deficiency of Calcitonin Gene-Related Peptide Affects Macrophage Polarization in Osseointegration

**DOI:** 10.3389/fphys.2020.00733

**Published:** 2020-07-31

**Authors:** Ying Yuan, Yixuan Jiang, Bin Wang, Yanjun Guo, Ping Gong, Lin Xiang

**Affiliations:** State Key Laboratory of Oral Diseases, National Clinical Research Center for Oral Diseases, Department of Oral Implantology, West China Hospital of Stomatology, Sichuan University, Chengdu, China

**Keywords:** bone remodeling/regeneration, cell biology, dental implant(s), immunity, histochemistry, cytokine(s)

## Abstract

Macrophages have been described as a critical cell population regulating bone regeneration and osseointegration, and their polarization phenotype is of particular importance. Several studies have shown that calcitonin gene-related peptide-α (CGRP) might modulate macrophage polarization in inflammatory response and bone metabolism. This study aimed to investigate the effect of CGRP on macrophage polarization in titanium osseointegration. ***In vitro***, bone marrow-derived macrophages (BMDMs) from C57BL/6 or CGRP**^–^**^/^**^–^** mice were obtained and activated for M1 and M2 polarization. Flow cytometry and real-time PCR were used to evaluate the M1/M2 polarization and inflammatory function. ***In vivo***, mice were divided into 3 groups: wild-type, CGRP**^–^**^/^**^–^**, and CGRP**^–^**^/^**^–^** mice with CGRP lentivirus. After extraction of the maxillary first molar, 0.6 mm **×** 1.25 mm titanium implants were emplaced. Bone formation and inflammation levels around implants were then observed and analyzed. The results of flow cytometry demonstrated that CGRP deficiency promoted M1 polarization and inhibited M2 polarization in BMDMs, which was consistent with pro-inflammatory and anti-inflammatory cytokine expression levels in real-time PCR. ***In vivo***, compared with the CGRP**^–^**^/^**^–^** group, the CGRP gene transfection group displayed better osseointegration and lower inflammation levels, close to those of the wild-type group. These results revealed that CGRP might play roles in macrophage polarization. In addition, CGRP deficiency could inhibit osseointegration in murine maxillae, while CGRP recovery by lentivirus transfection could improve osseointegration and regulate macrophage phenotype expression.

## Introduction

Dental implants have been widely employed as an effective treatment for edentulous conditions. The goal of many dental and orthopedic implants is the complete and direct integration of the implant and bone, in other words, osseointegration (Brown and Badylak, 2013; Viola et al., 2019). Osseointegration results from functional coupling and equilibrium not only between osteoblasts and osteoclasts but also between bone tissue and the immune system (Lee and Bance, 2019). Activation of the immune system could regulate the initial host response to the implant and affect its long-term survival. In this process, immune cells can release cytokines to modulate the microenvironment and immune response at the bone-implant surface (Hotchkiss et al., 2016).

Over several decades, the role of macrophages in bone metabolism and osseointegration has been brought into focus. Polarization phenotypes of macrophages exist on a broad spectrum from “classically activated” M1 to “alternatively activated” M2 macrophages (Viola et al., 2019). M1 and M2, known as the two representative and typical phenotypes, can secrete various cytokines in different tissue microenvironments (Mantovani et al., 2004). M1 macrophages synthesize interleukin (IL)-1b, IL-6, IL-12, IL-23, tumor necrosis factor-alpha (TNF-α), interferon-gamma (IFN-γ), monocyte chemoattractant protein 1 (MCP-1), macrophage inflammatory protein 1 beta (MIP-1β), and inducible nitric oxide synthase (iNOS) to boost inflammation and strengthen tissue defense (Yang et al., 2019). Identified as M2a, M2b, M2c, and M2d via different activators and biological behavior, M2 macrophages generally produce IL-1Ra, IL-10, and arginase-1(Arg1), as well as transforming growth factor (TGF)-β, which can support the homing, proliferation, and osteogenic differentiation of bone marrow-derived mesenchymal stem cells (BMSCs; Klopfleisch, 2016). Therefore, the balance of M1/M2 polarization of macrophages has been recognized to play key roles in regulating bone formation and implant osseointegration.

Calcitonin gene-related peptide-α (CGRP), the 37-amino acid neuropeptide secreted chiefly by peripheral nerve fibers, participates in the regulation of algesia, vessel formation, and immunity. Recently, several studies have shown that CGRP has important effects on bone remodeling (Naot and Cornish, 2008). Our previous study also found that CGRP deficiency led to a decreased level of osseointegration, while the recovered expression of CGRP at implant sites could promote bone healing and angiogenesis around implants in murine femurs (Xiang et al., 2017; Wang T. et al., 2018). Moreover, as a highly expressed sensory signal, CGRP is an essential member of the neuro-immune communication network, playing multifunctional roles at different sites by binding to its receptors, calcitonin receptor like-receptor (CRLR) and receptor activity-modifying protein 1 (RAMP1), which have been found to be expressed on different cells such as macrophages and BMSCs (Fernandez et al., 2001). Nevertheless, the exact function of CGRP on macrophage polarization in the osseointegration interface remains unclear.

Most studies use femur implants in the murine model to understand the biological basis of osseointegration. However, bone formation in long/endochondral bones is achieved through the program of endochondral ossification, which differs from that occurring in maxillary/mandibular bone (Sarem et al., 2018). Besides, there is a large proportion of marrow cavity in the implant sites of long bones, leading to a slower reaction to implant placement and ossification compared to the periosteum region (Cha et al., 2015). Thus, we proposed to place implants at teeth extraction sockets of maxillary first molars to simulate human dental implants.

The objective of this study is to investigate the effect of CGRP on macrophage polarization in osseointegration around dental implants so as to explore the pleiotropic effect of CGRP in bone metabolism.

## Materials and Methods

### Animals

All animal care and studies were approved by the Animal Research Committee of Sichuan University (WCHSIRB-D-2017-172, Chengdu, China) and were conducted following international standards. Male CGRP**^–/–^** mice (knockout model) and CGRP**^+/+^** mice (wild-type model) at the age of 8 weeks old with the same background were purchased from RIKEN BioResource Center (Tokyo, Japan). All animals were fed with standard diet *ad libitum* and housed under climate-controlled conditions.

### Isolation and Culture of Bone Marrow-Derived Macrophages

Primary bone marrow-derived macrophages (BMDMs) from knockout mice and wild-type mice were isolated and differentiated using standard protocols (Ying et al., 2013). Primary macrophages were derived from bone marrow cells and cultured for 7 days in RPMI-1640 (Gibco, Grand Island, NY, United States)-containing macrophage colony-stimulating factor (M-CSF; 10 ng/mL, Peprotech, 315-02, United States) and 10% fetal bovine serum (FBS; Gibco, Grand Island, NY, United States). At day 7, cells were collected by cell scraper for identification and polarization. Bone marrow-derived macrophages were divided into three groups: the knockout group, knockout with recombinant CGRP supplement (1 × 10^–8^ mol/L, Phoenix Pharmaceuticals, United States) group, and wild-type group. Each group contained 11 animals. For M1 activation, RPMI 1640 containing 10% FBS, 100 ng/mL LPS (Sigma, L2880, St. Louis, MO, United States) and 20 ng/mL IFN-γ (Gibco, PMC4033, Grand Island, NY, United States) was used; for M2 activation, RPMI 1640 containing 10% FBS with 25 ng/mL IL-4 (Peprotech, 214-14, United States) and 50 ng/mL IL-13 (Peprotech, 210-13, United States) was used. Real-time PCR and flow cytometry tests were applied at different time points.

### Flow Cytometry Tests

Macrophage phenotypes were analyzed by flow cytometry. Cells were suspended in 2% FBS/PBS, preincubated with anti-CD16/32 antibody (BioLegend, 101325, San Diego, CA, United States) to prevent non-specific binding via FcRII/III interactions, then incubated with anti-mouse antibody (F4/80-FITC,123107; CD11b-PE, 101207; CD86-PE, 159203; and CD206-FITC, 141703; BioLegend, San Diego, CA, United States). All samples were analyzed in triplicate. The analysis was performed on the LSR II Analyzer (BD Immunocytometry Systems, San Diego, CA, United States) in the Stanford Shared FACS Facility. FlowJo software (TreeStar, Ashland, OR, United States) was used for analysis. All samples were analyzed in triplicate.

### Implant Surgery

Calcitonin gene-related peptide-α overexpression lentiviral vector system was constructed and applied as described previously (Xiang et al., 2017). The lentivirus was used with Enhancer reagent and 5 μg/ml polybrene (GENECHEM Co., Shanghai, China). Animals were divided into the following three groups: (1) KO group: CGRP**^–^**^/^**^–^** mice without injection; (2) transfection group: KO mice with CGRP lentiviral vector injection into the prepared sites in the tooth extraction socket before implant insertion; (3) WT group: CGRP^+/+^ mice without injection. Each group contained 20 animals. At 6 weeks old, the bilateral maxillary first molars were extracted from all groups. In the palatal root socket, a φ0.5 mm pilot drill with a low-speed dental engine was used to create an osteotomy. The transfection group was injected with 10 μL CGRP lentiviral vector (5 × 10^8^TU/mL) into the prepared implant beds. The titanium implants (pure titanium, grade 4, SLA surface modification, diameter: 0.6 mm, length: 1.25 mm; WEGO, Shandong Province, China) were emplaced immediately following tooth extraction.

### Total RNA Isolation and Real-Time PCR

From the *in vitro* and *in vivo* tests, total RNA was extracted using Trizol Reagent (Invitrogen, Carlsbad, CA, United States) from each sample. From the *in vivo* tests, animals were sacrificed at day 7 and day 14, and maxillary bone with the implant (1 mm distal and mesial to the implant site) was carefully dissected without soft tissues. The bone tissues were then snap-frozen in liquid nitrogen, and the implant was removed. The RNA was treated with DNase, and then cDNA was synthesized using PrimeScript Reverse Transcriptase (Takara Bio, Inc., Shiga, Japan). Real-time PCR was conducted in triplicate in a 20-μL reaction mixture and was performed using an ABI PRISM 7300 Real-Time PCR System. Calculations of relative mRNA expression levels were performed according to the 2^–Δ^ ^Δ^ ^Ct^ method and were presented as fold increase relative to the control group. The primer sequences are shown in Table 1.

**TABLE 1:** Primer sequences for RT-qPCR.

Primer	Forward primer 5’–3’	Reverse primer 5’–3’
CD86	TCAATGGGACTGCATATCTGCC	GCCAAAATACTACCAGCTCACT
TNF-α	CAGGCGGTGCCTATGTCTC	CGATCACCCCGAAGTTCAGTAG
iNOS	GTTCTCAGCCCAACAATACAAGA	GTGGACGGGTCGATGTCAC
IL-1β	GAAATGCCACCTTTTGACAGTG	TGGATGCTCTCATCAGGACAG
CD206	CTCTGTTCAGCTATTGGACGC	TGGCACTCCCAAACATAATTTGA
Arg1	CTCCAAGCCAAAGTCCTTAGAG	GGAGCTGTCATTAGGGACATCA
PPAR-γ	GGAAGACCACTCGCATTCCTT	GTAATCAGCAACCATTGGGTCA
TGF-β	GAGCCCGAAGCGGACTACTA	TGGTTTTCTCATAGATGGCGTTG
BSP	GGAGGGGGCTTCACTGAT	AACAATCCGTGCCACCA
RUNX2	GAGGCCGCCGCACGACAACCG	CTCCGGCCCACAAATCTCAGA
CGRP	AGATGAAAGCCAGGGAGCTG	AGGTCTTGTGTGTACGTGCC
GFP	GACGACGGCAACTACAAGAC	TTCTGCTTGTCGGCCATGATA
GAPDH	AAGGCCGGGGCCCACTTGAA	GGACTGTGGTCATGAGCCCTTCCA

### Identification of CGRP-GFP Lentiviral Vector Transfection

To investigate whether the CGRP-GFP lentiviral vector system was successfully transfected into peri-implant sites, the IVIS Spectrum imaging system (PerkinElmer, Inc.) (absorbance of 465 nm), immunofluorescence labeling of GFP (Abcam, ab1218), and real-time PCR were applied to observe the expression of GFP in the transfection group at day 28 after local lentivirus injection. All samples were analyzed in triplicate.

### Micro-CT Measurement

The undecalcified samples of maxillary bone at day 14 and 28 were scanned using micro-CT (SCANCO 50, Switzerland) at 7-μm resolution. Exposure parameters were set at 90 kV and 200 μA. A volume of interest (VOI) was established as a radius of 20 μm around implants to define the peri-implant region. The morphometric analysis was performed by the evaluation script of bone tissue: bone volume fraction (BV/TV) and bone-implant-contact (BIC). All samples were analyzed in triplicate.

### Immunohistochemical Analysis

Three maxillary bones of each group were decalcified at day 14 and dehydrated in the condition of ascending concentration of ethanol (70–95%) then cleared with xylene and embedded in a paraffin block. Three 5-μm thick longitudinal sections were obtained from each sample. Sections were immunostained for iNOS (Santa Cruz, sc-271430, United States) and Arg1(BD, 610328, United States).

### Statistical Analysis

Statistical analysis was performed with SPSS 17.0. All assays were performed in triplicate, and each experiment was repeated at least three times. The results were presented as mean ± SD. Data were analyzed by ANOVA using a multiple comparison Dunnett *post hoc* test. *P* < 0.05 was considered statistically significant.

## Results

### Identification of Bone Marrow-Derived Macrophages by Flow Cytometry

**FIGURE 1:**
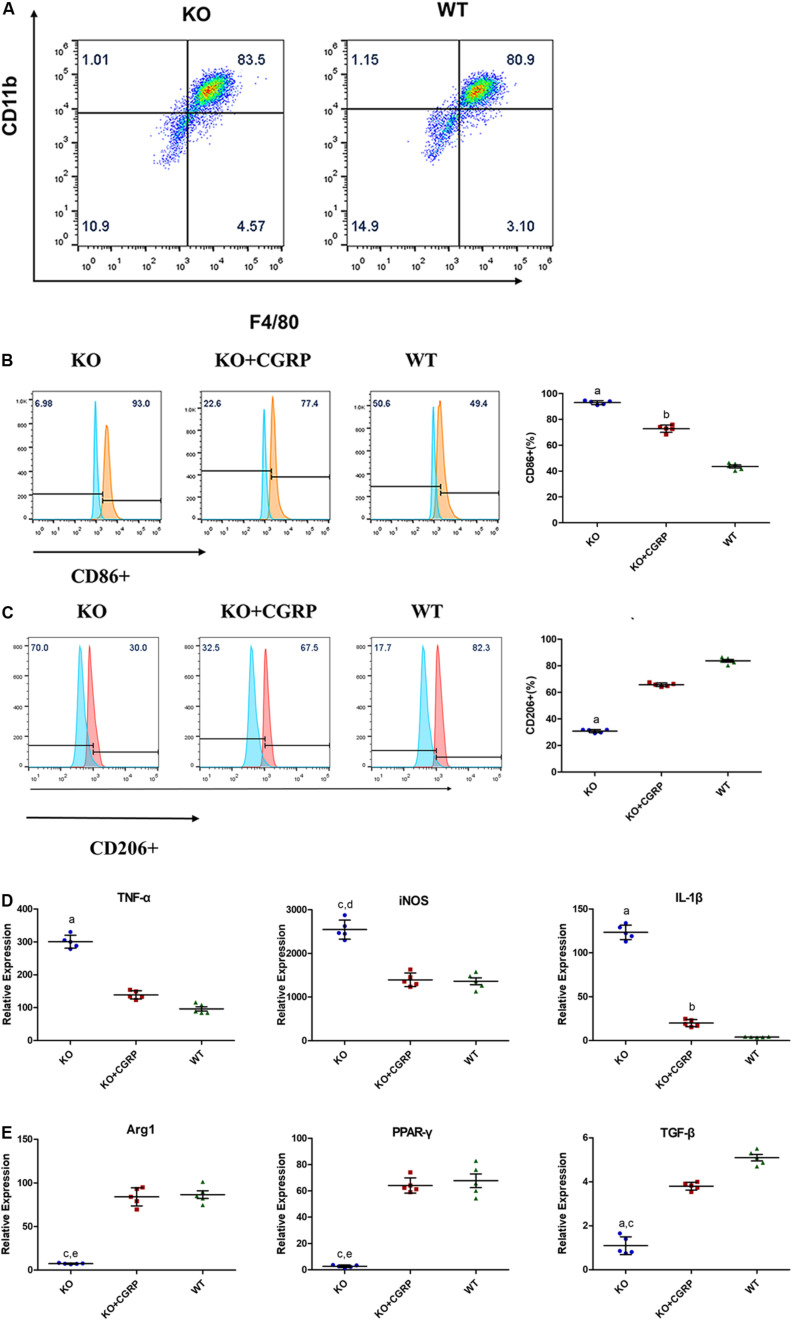
Flow cytometry analysis showed that there were 83.3 + 0.82% CD11b+ and F4/80+ cells in the KO group and 81.60 + 1.11% in the WT group at day 7 **(A)** (*n* = 3). Levels of CGRP regulated the polarization of macrophages. CGRP deficiency enhanced CD86 **(B)** in M1 polarization and inhibited CD206 **(C)** in M2 polarization, according to flow cytometry analysis. Relative expressions of TNF-α, iNOS, and IL-1β in LPS/IFN-γ-induced BMDMs **(D)** were upregulated in the CGRP-/- group, while CGRP supplements could inhibit M1 expression. As for the IL-4/IL-13 induced group **(E)**, CGRP deficiency inhibited the expression of Arg1, TGF-β, and (PPAR-γ). CGRP supplement could restore their expression levels. *n* = 5 specimens/group. a: *P* < 0.001, for KO vs. others; b: *P* < 0.01, for KO + CGRP vs. WT; c: *P* < 0.05, for KO vs KO + CGRP; d: *P* < 0.01, for KO vs WT; e: *P* < 0.01, for KO vs KO + CGRP.

At day 7, mature BMDMs were evaluated by flow cytometry analysis and fluorophore-conjugated antibodies to detect the expression of F4/80 and CD11b. There were over 80% CD11b^+^ and F4/80^+^ cells in both the WT and KO groups on day 7 (Figure 1A).

### The Effect of CGRP on BMDM Polarization

Flow cytometry and real-time PCR were conducted to characterize macrophage polarization. We found that CGRP deficiency enhanced the expression of the M1 surface marker CD86 (Figure 1B) and inhibited the expression levels of CD206 (Figure 1C). The results demonstrated that in lipopolysaccharide (LPS)/IFN-γ-induced groups, CGRP deficiency significantly enhanced the relative expression of TNF-α, iNOS, and IL-1β gene in mRNA levels, while CGRP supplement could inhibit M1 expression (Figure 1D). As for IL-4/IL-13 induced groups, CGRP deficiency inhibited the expression of Arg1, TGF-β, and peroxisome proliferator-activated receptor-γ (PPAR-γ). Exogenous CGRP supplement could restore their expression levels (Figure 1E).

### Identification of CGRP-GFP Lentiviral Vector Transfection *in vivo*

**FIGURE 2:**
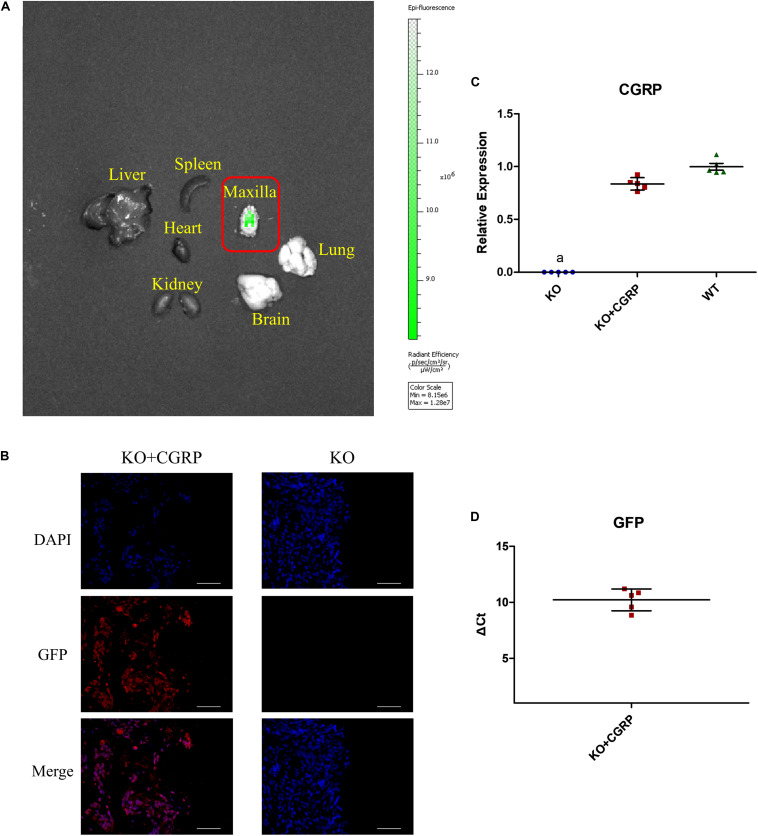
Transfection verified via the IVIS system, immunofluorescence, and real-time PCR. The IVIS Spectrum imaging system found that there was obvious GFP-labeled CGRP transgene expression at the peri-implant site on 28 days, which demonstrated successful target-gene transfection into CGRP-/- mice **(A)**. Immunofluorescence also proved positive expression of GFP **(B)**. Real-time PCR also revealed the obvious expression of CGRP and GFP at the implantation sites in the transfection group after 28 days **(C,D)**. *n* = 5 specimens/group. a: *P* < 0.001, for KO vs. others. Scale bar = 50 μm.

The IVIS Spectrum imaging system was applied to verify the expression of CGRP around titanium implants 28 days after the operation. There was a prominent GFP-labeled CGRP transgene expression at the peri-implant site, which indicated successful target-gene transfection into KO mice (Figure 2A). The immunofluorescence proved that GFP expressed around implants in the KO + CGRP group (Figure 2B). Real-time PCR also revealed that the positive mRNA expression of CGRP and GFP were regained at the implantation sites in the transfection group after 28 days (Figures 2C,D).

### The Impact of CGRP Expression Levels on Osseointegration

**FIGURE 3:**
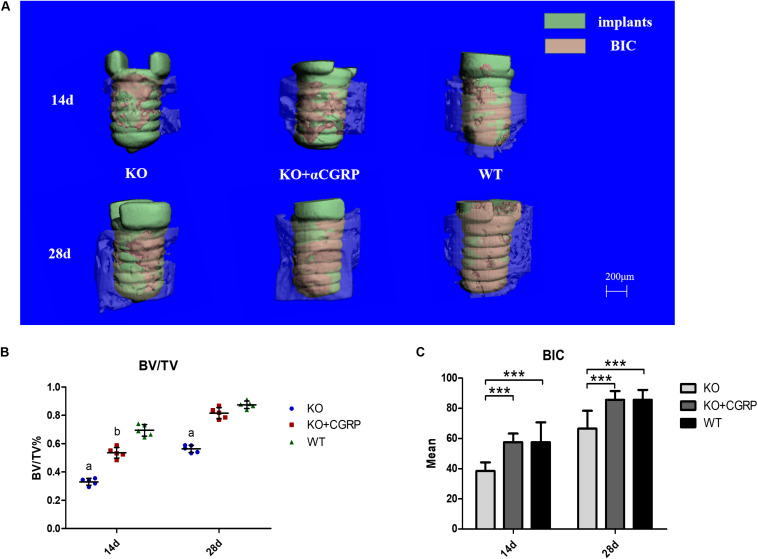
The impact of CGRP expression levels on osseointegration according to micro-CT tests. From the three-dimensional reconstruction of the implant, deficiency of CGRP impeded bone-implant contact **(A)**. The quantitative analysis of micro-CT showed that transfection with CGRP enhanced the expression level of BV/TV **(B)** and BIC **(C)** compared to the KO group, while these bone markers were approximate to the WT group. *n* = 5 specimens/group. a: *P* < 0.001, for KO vs. others; b: *P* < 0.05, for KO + CGRP vs. WT. d: *P* < 0.01, for KO vs. WT; e: *P* < 0.05, for KO vs. KO + CGRP. ***P < 0.001.

From the three-dimensional reconstruction of the implant, we found that deficiency of CGRP impeded bone-implant contact (Figure 3A). Quantitative analysis of micro-CT was then performed, and the results suggested that transfection with CGRP enhanced the expression level of BV/TV and BIC compared to the KO group. At the same time, these bone markers were similar to the WT group (Figures 3B,C). These results elucidated that CGRP was involved in the regulation of osseointegration around dental implants.

### CGRP Regulated Osteogenic Gene Expression

**FIGURE 4:**
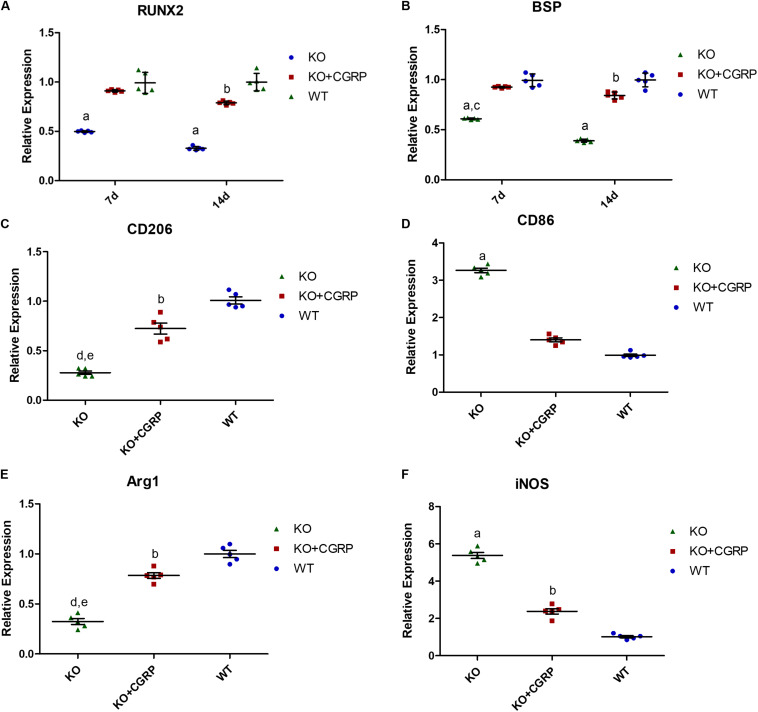
Relative expression levels of Runx2 **(A)**, BSP **(B)**, CD206 **(C)**, CD86 **(D)**, Arg1 **(E)** and iNOS **(F)**. Expression levels of Runx2 and BSP were higher in the KO + CGRP group compared to the KO group. There was no significant difference between the transfection group and the WT group at each time point (*P* > 0.05). At 7 days, the relative expressions of CD206 and Arg1 were inhibited and those of CD86 and iNOS were enhanced in the KO group. There is no significant difference between the KO + CGRP group and the WT group. *n* = 5 specimens/group. a: *P* < 0.001, for KO vs. others; b: *P* < 0.05, for KO + CGRP vs. WT. c: *P* < 0.01, for KO vs. KO + CGRP. c: *P* < 0.01, for KO vs. KO + CGRP.

From day 14 to day 28, expression levels of Runx2 and BSP were higher in the transfection group compared to the KO group. There was no significant difference between the transfection group and the WT group at each time point (*P* > 0.05) (Figures 4A,B). The results also revealed that CGRP regulated osteogenic markers in this model.

### The Effects of CGRP on the Recruitment of Macrophages With Different Subtypes *in vivo*

**FIGURE 5:**
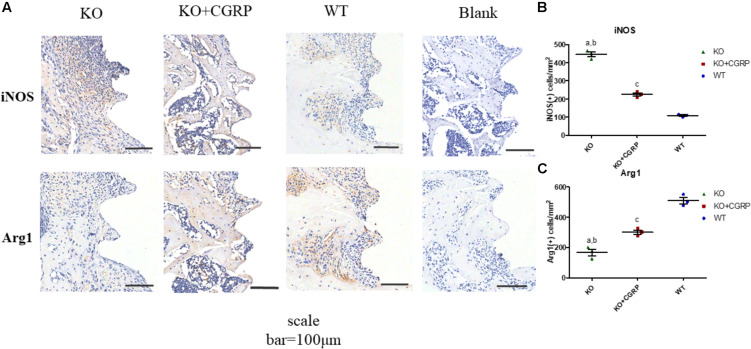
IHC staining of iNOS and Arg1 around implants. Both iNOS-positive cells and Arg1-positive cells could be found in the bone tissues around the implants **(A)**. IHC staining and quantitative analysis for iNOS **(B)** and Arg1 **(C)** are shown. Data are presented as mean standard deviation (SD), *n* = 3 specimens/group. a: *P* < 0.001, for KO vs. WT; b: *P* < 0.01, for KO vs. KO + CGRP. c: *P* < 0.001, for KO + CGRP vs. WT.

As for the phenotype of macrophages, real-time PCR analysis illuminated that transfection with CGRP could enhance the expression levels of M2 markers (Arg1 and CD206) and inhibited M1 markers (CD86 and iNOS) compared to the KO group (Figures 4C–F) at day 7. According to the results of IHC staining, both iNOS-positive cells and Arg1-positive cells could be found in the bone tissues around implants. iNOS-positive macrophages in the transfection group were much lower than in KO mice; Arg1-positive cells were the opposite. There was no significant difference between the WT group and the transfection group (Figure 5).

## Discussion

The monocyte/macrophage is one of the most important cell types of the immune system. Pro-inflammatory M1 macrophages are induced by IFN-γ or LPS and generally express IL-1β, IL-6, iNOS, and TNF-α with surface markers CD86 and CCR7 (Shapouri-Moghaddam et al., 2018). In contrast, anti-inflammatory M2 macrophages are induced by IL-4/IL-13, IL-10, and immune complexes (IC). M2 activated macrophages produce high levels of Arg1 and IL-10 with surface markers CD163 and mannose receptor (CD206) (Horwood, 2016). Previous studies indicated that nerve and immune cells could secrete CGRP in response to particular stimuli, such as temperature, immune response, and tissue injury (Monneret et al., 2003; Tepper, 2018). Feng et al. (1997) found that LPS-induced TNF-α production could be inhibited by CGRP via cAMP response in mouse macrophages. In this study, we found that CGRP deficiency promoted the LPS/IFN-γ-induced classical activation of macrophages, with increased expression of TNF-α, IL-1β, iNOS, and CD86. Meanwhile, IL-4/IL-13-induced alternative activation of macrophages was suppressed, which was manifested by decreased expression of Arg1, IL-10, TGF-β, and CD206. However, recombinant CGRP supplements could recover these effects. These findings suggested that CGRP could regulate the polarization of macrophages.

Recently, an increasing body of evidence has demonstrated that an appropriate immuno-microenvironment is essential for successful osseointegration (Brown and Badylak, 2013; Loi et al., 2016). The balance between pro- and anti-inflammatory mediators dictated the activation and resolution of inflammation (Duan et al., 2017). However, there was a debate about whether the M1 or M2 phenotype increased MSC osteogenic differentiation *in vitro* (Guihard et al., 2012; Nicolaidou et al., 2012; Gong et al., 2016). Pajarinen et al. reported that M1 macrophages might promote the early and middle stages of osteogenesis, while M2 macrophages contributed to matrix mineralization much later (Pajarinen et al., 2019). Appropriate switching from M1 to M2 phenotype might be crucial for bone fracture healing and implant osseointegration (Claes et al., 2012; Wang J. et al., 2018; Medhat et al., 2019). In this study, IHC staining indicated that both iNOS-positive cells and Arg1-positive cells could be found around the implants. The number of iNOS-positive macrophages was the highest in the three groups, while the number of Arg1-positive cells was the lowest, suggesting that more M1 macrophages infiltrated in the CGRP**^–^**^/^**^–^** group and more M2 macrophages in the CGRP^+/+^ group and transfection group in the early stage of osseointegration. Compared to the KO group, CGRP^+/+^ is prone to induce more macrophages to the M2 phenotype, which might be beneficial to wound healing and osseointegration around dental implants.

Previous research revealed that CGRP might be involved in many physiological and pathophysiological events, such as chronotropic and inotropic actions in the heart, dilatation of arterial vessels, relaxation of urinary smooth muscle, and immunoreaction response (Russell et al., 2014; Kuzawińska et al., 2016). In the process of bone metabolism, CGRP could regulate osteoblasts and osteoclasts directly and indirectly (Tuzmen and Campbell, 2018; Jia et al., 2019). Yoo et al. (2014) confirmed that CGRP could inhibit bone resorptive activities through the RANKL/OPG pathway and induce osteoblast differentiation via canonical Wnt signaling. Besides, CGRP could be an important regulator in fracture healing through influencing phosphorylated ERK expression (Duan et al., 2017). Our work illuminated that CGRP might improve implant osseointegration by regulating macrophage polarization.

Gene engineering has been widely used to explore the exact role of a gene and to observe the corresponding changes of downstream signaling molecules. To investigate the role of CGRP in the early stage of osseointegration, we successfully constructed CGRP gene knockout mice and the CGRP lentivirus vector and established a CGRP rescue model at the peri-implant site of mouse femur in our previous study (Xiang et al., 2017). In the present study, a mouse model with implants placed in the maxillary first molar extraction sockets was established, and lentivirus vector was used in the same way in the oral environment. Our results confirmed that CGRP depletion had an adverse impact on bone formation around implants in the maxilla alveolar bone, which was in accordance with the previous results for femur implants in mice.

Several studies have indicated that CGRP might regulate macrophage polarization and inhibit inflammation in murine macrophages (Duan et al., 2017). Consistently, with the mouse model of maxillary implants and the consequent histological analysis, our research also demonstrated that CGRP played a crucial role in the osteogenesis/osseointegration and macrophage polarization. CGRP recovery with lentivirus transfection could improve osseointegration and modulate inflammation infiltration continuously and effectively.

## Conclusion

Our study suggested that CGRP might play an important role in macrophage polarization. CGRP deficiency impeded the implant osseointegration in murine maxillae, while CGRP recovery with lentivirus transfection could improve osseointegration and regulate macrophage phenotype expression. The exact mechanism of macrophage polarization in osseointegration remains an intriguing topic; it may correlate with the mesenchymal stem cell-macrophage crosstalk and will be one of the focuses of our future studies.

## Data Availability Statement

The datasets generated for this study are available on request to the corresponding author.

## Ethics Statement

The animal study was reviewed and approved by the Animal Research Committee of Sichuan University.

## Author Contributions

YY contributed to the conception, design, and analysis, drafted the manuscript, and critically revised the manuscript. YJ and BW contributed to the conception, data acquisition, and interpretation and critically revised the manuscript. YG contributed to the analysis and drafted the manuscript. PG and LX contributed to the conception and design and critically revised the manuscript. All authors gave final approval and agreed to be accountable for all aspects of the work.

## Conflict of Interest

The authors declare that the research was conducted in the absence of any commercial or financial relationships that could be construed as a potential conflict of interest.
